# Systematic identification of DNA variants associated with ultraviolet radiation using a novel Geographic-Wide Association Study (GeoWAS)

**DOI:** 10.1186/1471-2350-14-62

**Published:** 2013-06-20

**Authors:** Irving Hsu, Rong Chen, Aditya Ramesh, Erik Corona, Hyunseok Peter Kang, David Ruau, Atul J Butte

**Affiliations:** 1 Division of Systems Medicine, Department of Pediatrics, Stanford University School of Medicine, 1265 Welch Road, MS-5415, Stanford, CA 94305, USA; 2 Lucile Packard Children’s Hospital, 725 Welch Road, Palo Alto, CA 94304, USA; 3 Irvington High School, 41800 Blacow Road, Fremont, CA 94538, USA; 4 Biomedical Informatics Graduate Training Program, Stanford, CA 94305, USA; 5 Personalis, Inc, 1350 Willow Road, Suite 202, Menlo Park, CA 94025, USA

## Abstract

**Background:**

Long-term environmental variables are widely understood to play important roles in DNA variation. Previously, clinical studies examining the impacts of these variables on the human genome were localized to a single country, and used preselected DNA variants. Furthermore, clinical studies or surveys are either not available or difficult to carry out for developing countries. A systematic approach utilizing bioinformatics to identify associations among environmental variables, genetic variation, and diseases across various geographical locations is needed but has been lacking.

**Methods:**

Using a novel Geographic-Wide Association Study (GeoWAS) methodology, we identified Single Nucleotide Polymorphisms (SNPs) in the Human Genome Diversity Project (HGDP) with population allele frequencies associated geographical ultraviolet radiation exposure, and then assessed the diseases known to be assigned with these SNPs.

**Results:**

2,857 radiation SNPs were identified from over 650,000 SNPs in 52 indigenous populations across the world. Using a quantitative disease-SNP database curated from 5,065 human genetic papers, we identified disease associations with those radiation SNPs. The correlation of the rs16891982 SNP in the *SLC45A2* gene with melanoma was used as a case study for analysis of disease risk, and the results were consistent with the incidence and mortality rates of melanoma in published scientific literature. Finally, by analyzing the ontology of genes in which the radiation SNPs were significantly enriched, potential associations between SNPs and neurological disorders such as Alzheimer’s disease were hypothesized.

**Conclusion:**

A systematic approach using GeoWAS has enabled us to identify DNA variation associated with ultraviolet radiation and their connections to diseases such as skin cancers. Our analyses have led to a better understating at the genetic level of why certain diseases are more predominant in specific geographical locations, due to the interactions between environmental variables such as ultraviolet radiation and the population types in those regions. The hypotheses proposed in GeoWAS can lead to future testing and interdisciplinary research.

## Background

Over the past decade, high-throughput technologies and advancements in computational biology have made it possible to determine genotypes and complete human DNA sequences at a continued reduction of cost, and these technologies have been used to determine numerous variants associated with various diseases and traits. Why and when variants leading to disease susceptibility entered the human genome remains unclear, but could be elucidated through the study of genetics in indigenous populations, more closely representing original human subpopulations across the world [1]. How these disease variants might be -- or have been -- associated with environmental conditions might yield insights into why those disease variants are present in the genome.

To study early human migrations, in 2005, Li et al. analyzed 1064 individuals from indigenous populations at over 650,000 Single Nucleotide Polymorphisms (SNPs) in the Human Genome Diversity Project (HGDP) [2,3]. The HGDP collection is currently the most comprehensive human DNA collection representing the world’s population distribution available to not-for-profit researchers. Unlike genome-wide association studies (GWAS) [4], which were performed mostly on populations of European ancestry, human genome databases from the HGDP have enabled biomedical researchers to study how the genetic risks of different diseases vary over a global range of ethnic populations [2]. The availability of individual level genotypes across various countries and populations has led to an increased interest in understanding how human diseases are associated with environmental variables, since these variables are usually geographically dependent. Moreover, many diseases are understood to result from the complex interactions between both genetic and environmental factors.

One important environmental factor is ultraviolet (UV) radiation, which has been linked to a wide range of human diseases. Interactions between skin pigmentation genes and UV radiation have been previously studied, but only on a country-wide scale [5,6]. Using SNPs pre-selected from genes of interest, these works investigated the role of pigmentation in melanoma predisposition within single countries, Spain and Australia, respectively. SNPs associated with UV radiation have not been identified across the world by using a systematic approach, but such a world-wide study carries obvious difficulties [2].

In another recent study [7], evidence was found for human adaptations to climate at the genome-wide level. This was accomplished by identifying the SNPs with allele frequencies that were the most strongly correlated with several different climate variables across the world. Among the phenotypes that were found to be associated with these SNPs, however, the number of diseases identified was limited. Only one disease – Systemic lupus erythematosus (SLE) – was reported to be significantly correlated with solar radiation.

In this paper, we investigate UV radiation and its effects at the genome-wide level across a wider range of human diseases in different countries. Human genomes from 52 native populations in the HGDP panel were mapped to 193 different countries. We then designed a bioinformatics-based Geographic-Wide Association Study (GeoWAS) to systematically identify DNA variants that were the most strongly correlated with UV exposure levels across the world, which we called radiation SNPs. To identify diseases that are strongly associated with radiation SNPs, we queried these SNPs against a curated disease-SNP database called VARIMED (Variants Informing Medicine) [8,9]. With this developed methodology, we were able to investigate the effects and implications of UV radiation on human phenotypes and diseases at both the genomic and geographic levels. Lastly, the significance of GeoWAS and its promises are discussed from both biomedical and methodological perspectives.

## Methods

**Figure 1:**
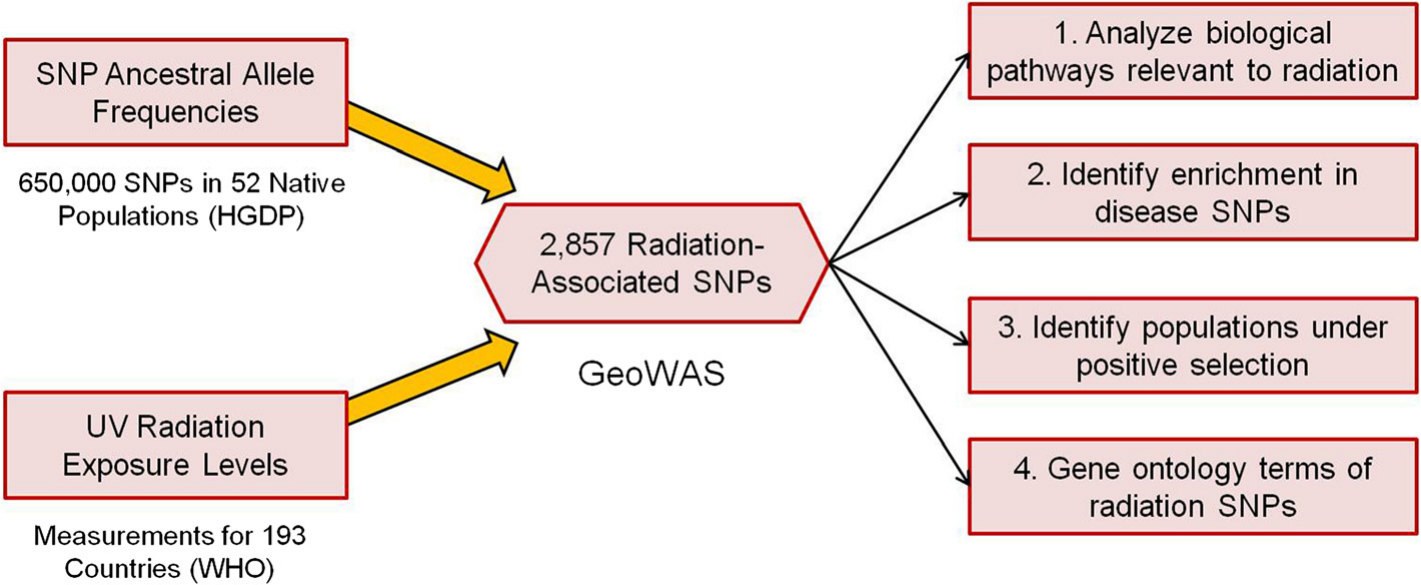
**GeoWAS methodology used to identify SNPs associated with UV radiation.** Once obtained, the radiation SNPs were further analyzed for positive selection in populations, enrichment in disease-related polymorphisms, and enrichment in human genes.

A Geographic-Wide Association Study (GeoWAS), outlined in Figure 1, was designed to analyze the associations between UV radiation exposure levels and the ancestral allele frequency of 650,000 SNPs in the genomes of 52 native populations across the world. These populations were genotyped under the Human Genome Diversity Project (HGDP) collection [2,3]. UV radiation data was obtained from the World Health Organization (WHO) environmental data repository [10].

**Figure 2:**
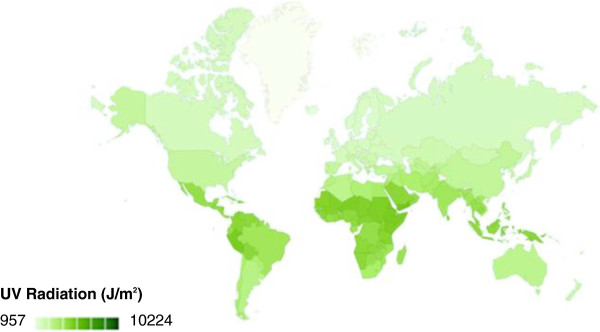
**UV radiation global distribution.** UV radiation levels are measured in Joules of ambient energy per square meter (J/m^2^). Darker hues correspond to higher levels of radiation.

To our knowledge, as of this writing, the WHO database has not yet been extensively used for genetic association studies. The radiation levels, given in joules of ambient UV energy per square meter (J/m^2^), are population-weighted averages, and are provided for each of 193 countries in the database. The radiation data was obtained from 1997 to 2003, and was assumed to remain at a constant level since its collection. We also assumed the UV radiation exposure was constant going back to the original establishment of the populations sampled in the HGDP. Using an interface between R and the Google Visualization API, we generated a map illustrating UV radiation exposure as a function of color for all of the countries across the world (Figure 2). We mapped each country to a corresponding HGDP population based on geographic proximity, and took into account both historical and cultural factors of the countries being mapped. In this study, it was important to use the native HGDP populations because they were already in place before the occurrence of the diasporas in the fifteenth and sixteenth centuries following the advent of sea navigation [2]. These movements contributed to genetic admixtures among different populations. The original populations from the HGDP panel are thus necessary to provide a control for complex genetic variations across the world resulting from these admixtures.

We analyzed 650,000 DNA variants in the form of single nucleotide polymorphisms (SNPs) for individuals from each of the 52 native populations. Each SNP has a corresponding ancestral allele and a derived allele produced by mutation events over time. The derived allele may become more common in human loci due to random genetic drift or selective pressures from the environment. For a given locus, the allele frequency represents the fraction of the chromosomes that carry the specified allele. For every SNP, the ancestral allele frequency (AAF) in each country was correlated with the radiation levels measured for those countries. Both Pearson’s and Spearman’s correlation coefficients and p-values were calculated to assess the relative strength of the associations. Each SNP was then annotated with its corresponding chromosome number, function type, gene symbol, and NCBI Gene ID [11].

These initial correlations served as a means of filtering the SNPs that we targeted for further analysis. We identified SNPs that had ancestral allele frequencies significantly associated with UV radiation exposure by focusing only on the SNPs with a Pearson’s absolute rho of greater than 0.8 (*r* > 0.8), a strict cutoff value determined from our preliminary permutation studies. These SNPs were classified as radiation SNPs.

### Validation using SNPs in Vitamin D synthesis pathway genes

To ascertain the effectiveness of our proposed methodology, we first examined biological processes known to be linked to radiation, and analyzed the degree to which these processes are supported by our approach. It is widely understood that variations in UV exposure influence the synthesis and metabolism of Vitamin D [12]. Six different genes (*CYP24A1*, *CYP27A1*, *CYP27B1*, *CYP2R1*, *DHCR7*, *GC*), taken from Reactome [13], were identified to be involved in Vitamin D synthesis pathways. The SNPs within these genes were compared with background SNPs from other genes in our database, and the degree of association was quantified.

### Analysis of diseases associated with radiation SNPs

After running our initial correlations, the radiation SNPs were queried against the VARIMED database to identify diseases and other phenotypes that the SNPs may be linked to. VARIMED is a quantitative human disease-SNP association database, curating from approximately 5500 different publications, as previously described [8].

### Populations under positive selection for radiation SNPs

The Integrated Haplotype Score (iHS) is a statistic for identifying recent positive selection at a particular locus, and is dependent upon differences in linkage disequilibrium (LD) around a positively selected allele compared to the background allele [14,15,17]. In all 52 HGDP populations, each of the 650,000 SNPs has a specific iHS score representing the degree of positive selection for it. We developed an algorithm that targets the populations most prone to selection for radiation SNPs, for further analysis. For each population, we mapped all of the available SNPs to their corresponding iHS scores, and calculated a selection metric for the set of radiation SNPs by taking the mean of the iHS scores. To obtain a relative measure of the significance of selection for the radiation SNPs, we computed empirical p-values by comparing the metric for each population against a null distribution of such metrics. In each population, the null distribution was generated by using arbitrary SNPs that were randomly selected among all SNPs for which data are available. For example, if UV radiation has *n* number of associated SNPs, then *n* samples were taken from the entire list of SNPs for a population. In this case, our null hypothesis for each population would be that it has not experienced selection for radiation SNPs. Those populations in which the empirical p-value is below a scalar alpha (α < 0.01) are considered to be under significant positive selection for the set of SNPs currently in question. We chose to implement this procedure using the HGDP data set, since we may view selection trends across a gamut of populations.

### Gene ontology terms of radiation SNPs

From the complete pool of 650,000 SNPs, those SNPs identified to be associated with UV radiation (radiation SNPs) were joined with their corresponding NCBI Entrez Gene IDs to create a gene list. From the list, we identified the genes in which the radiation SNPs were significantly enriched by joining the list with a table containing the Gene Ontology (GO) terms for these genes. We focused on the GO terms that had a p-value of under 0.05 after performing a Bonferroni correction. These genes and their potential connections to diseases were surveyed in published literature. Based on our findings, we formulated new hypotheses on how DNA variation in the genes of interest may be potentially related to UV radiation and diseases. These proposed mechanisms of disease formation can lead to future research and follow-up testing.

## Results

For each SNP measured in the Human Genome Diversity Project (HGDP), the ancestral allele frequency (AAF) in each of 193 countries was correlated with the radiation levels measured for those countries. After running these initial correlations, 2,857 of the 650,000 SNPs were identified to have AAFs significantly associated with UV radiation exposure, using a Pearson’s absolute rho of 0.8 as a strict cutoff value. These SNPs were designated as radiation SNPs, and each was annotated with its corresponding chromosome number, function type, gene symbol, and Entrez database Gene ID.

**Figure 3:**
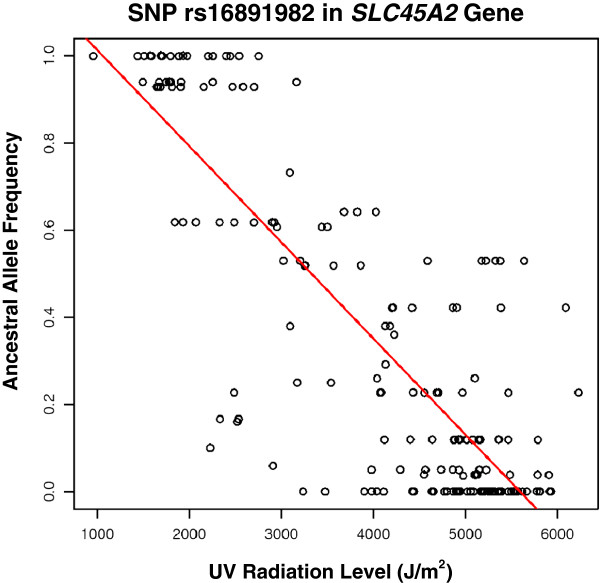
**Scatterplot correlating ancestral allele frequencies against the radiation levels in each country for the rs16891982 SNP.** Each point on the plot represents a single country. The rs16891982 SNP is found in the *SLC45A2* gene, and is known to be associated with phenotypes such as pigmentation and skin cancer. Specific country allele frequencies are shown in Figure 5.

The association between AAFs and radiation for each UV SNP can be represented as a scatterplot. As a specific example, the plot for the SNP rs16891982 is shown in Figure 3 (*r* = -0.831). A fairly strong negative relationship exists, with countries receiving greater levels of UV radiation having a lower SNP AAF. The rs16891982 SNP, which plays a critical role in pigmentation and in certain forms of skin cancer, will be emphasized in a case study discussed below.

### SNPs in Vitamin D synthesis pathway genes

To validate the efficacy of our methodology, we first evaluated genes involved in Vitamin D synthesis, known to be influenced by UV exposure. We curated six genes known to be involved in Vitamin D synthesis pathways, and conducted a Mann–Whitney U test to compare the Pearson p-values of the SNPs within these genes against those of background SNPs in other genes. In total, 155 different SNPs were contained in these six genes. We found that SNPs in genes for Vitamin D biosynthesis had a significant p-value in association with UV radiation (p = 3.0 × 10^-4^) than the set of background SNPs in other genes, indicating that the SNPs found using our methodology are relevant to pathways known to be associated with radiation.

### Radiation SNPs are significantly enriched for association with human diseases

Overall, 19 diseases and phenotypes from VARIMED were found to be associated with the 2,857 radiation SNPs (Table 1). Using a Hypergeometric test, these SNPs were determined to be significantly enriched for any disease association at 2.14 fold, compared with control genomic SNPs. The Pearson p-value for each disease-SNP pair indicates the published strength of the relation for each.

**Table 1:** Different phenotypes and diseases associated with radiation SNPs, identified through VARIMED

**Disease/Trait**	**SNP ID (rs)**	**R-value (Pearson)**	**Function type**	**Gene**	**Ancestral allele**	**P-min**
**Obesity**	10508503	0.841646	Intergenic	-	C	1.10 × 10^-7^
**Eye color**	10809808	0.800649	Intergenic	-	T	5.80 × 10^-12^
**Asthma**	10922300	0.805829	Intergenic	-	C	7.44 × 10^-11^
**Coffee consumption**	11856835	0.811979	Intron	*SEMA7A*	G	1.10 × 10^-7^
**HDL cholesterol levels**	1263173	0.815426	Intergenic	-	G	2.13 × 10^-7^
**Transferrin receptor levels**	1263173	0.815426	Intergenic	-	G	6.80 × 10^-14^
**Eye color**	12913832	0.801066	Intron	*HERC2*	A	1.00 × 10^-11^
**Hair color**	12913832	0.801066	Intron	*HERC2*	A	8.51 × 10^-10^
Basal cell carcinoma*	16891982	-0.83108	Missense	*SLC45A2*	G	1.60 × 10^-12^
**Eye color**	16891982	-0.83108	Missense	*SLC45A2*	G	1.48 × 10^-12^
**Hair color**	16891982	-0.83108	Missense	*SLC45A2*	G	3.89 × 10^-11^
Malignant melanoma*	16891982	-0.83108	Missense	*SLC45A2*	G	2.00 × 10^-8^
Melanoma*	16891982	-0.83108	Missense	*SLC45A2*	G	8.30 × 10^-15^
**Skin color**	16891982	-0.83108	Missense	*SLC45A2*	G	1.70 × 10^-9^
**Skin pigmentation**	16891982	-0.83108	Missense	*SLC45A2*	G	5.02 × 10^-8^
Squamous cell carcinoma*	16891982	-0.83108	Missense	*SLC45A2*	G	1.00 × 10^-7^
**Hair color**	28777	0.805006	Intron	*SLC45A2*	C	1.10 × 10^-8^
**Freckles**	2153271	0.800255	Intron	*BNC2*	C	3.98 × 10^-10^
**Metabolite traits**	2286963	0.813366	Missense	*ACADL*	T	3.10 × 10^-12^
**Coffee consumption**	2470893	-0.80281	Near Gene5	*CYP1A1*	T	2.90 × 10^-9^
**Amyloid beta-protein levels**	2899472	0.81232	Intron	*CYP19A1*	C	1.90 × 10^-12^
**Hair color**	3829241	0.831729	Missense	*TPCN2*	G	6.20 × 10^-14^
**HDL cholesterol levels**	496300	0.813658	Intergenic	-	T	3.90 × 10^-7^
**Hair color**	7196459	0.841939	Intron	*PRDM7*	G	3.10 × 10^-15^
Colorectal cancer*	7259371	0.812093	Intron	*RHPN2*	A	2.20 × 10^-7^
**Height**	798489	0.808614	Intron	*GNA12*	C	1.90 × 10^-8^
**Bone mineral density**	884205	0.806085	Intergenic	-	C	9.40 × 10^-9^

Empty values in the gene column indicate that the corresponding SNP is located outside of a gene region. Cancerous phenotypes are highlighted with an asterisk. As many of the same SNPs have been repeatedly identified for varying but similar diseases and conditions, these are not independent traits.

Of the 19 distinct phenotypes, 8 were associated with the rs16891982 SNP located in the *SLC45A2* gene. As shown in Table 1, this SNP was strongly associated with pigmentation and with various forms of skin cancer, including melanoma, basal cell carcinoma, and squamous cell carcinoma. The rs16891982 SNP is associated with each of these cancer types with the risk allele being *G*, while the ancestral allele is *G*, and derived allele is *C*. The correspondence between the risk allele and the ancestral allele has important implications on the skin cancers we identified. This indicates that native populations with higher ancestral allele frequencies are more susceptible to the skin cancers. Among these cancers, melanoma has been found to be significantly associated with rs16891982 through separate clinical studies previously conducted in Spain and Australia [5,6]. Although this is consistent with the results we obtained, such studies are confined to a single area and thus cannot be directly applied to make interregional comparisons.

**Figure 4:**
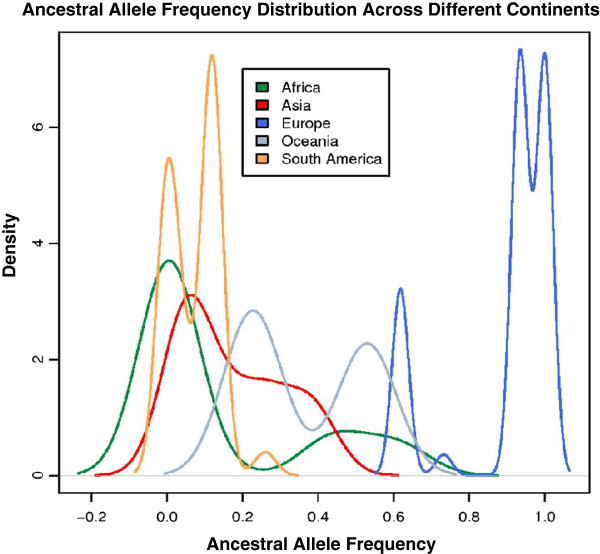
**Ancestral allele frequency distributions for rs16891982 across various HGDP continents.** For each continent, the distribution was calculated by aggregating the allele frequencies of all the countries within it.

**Figure 5:**
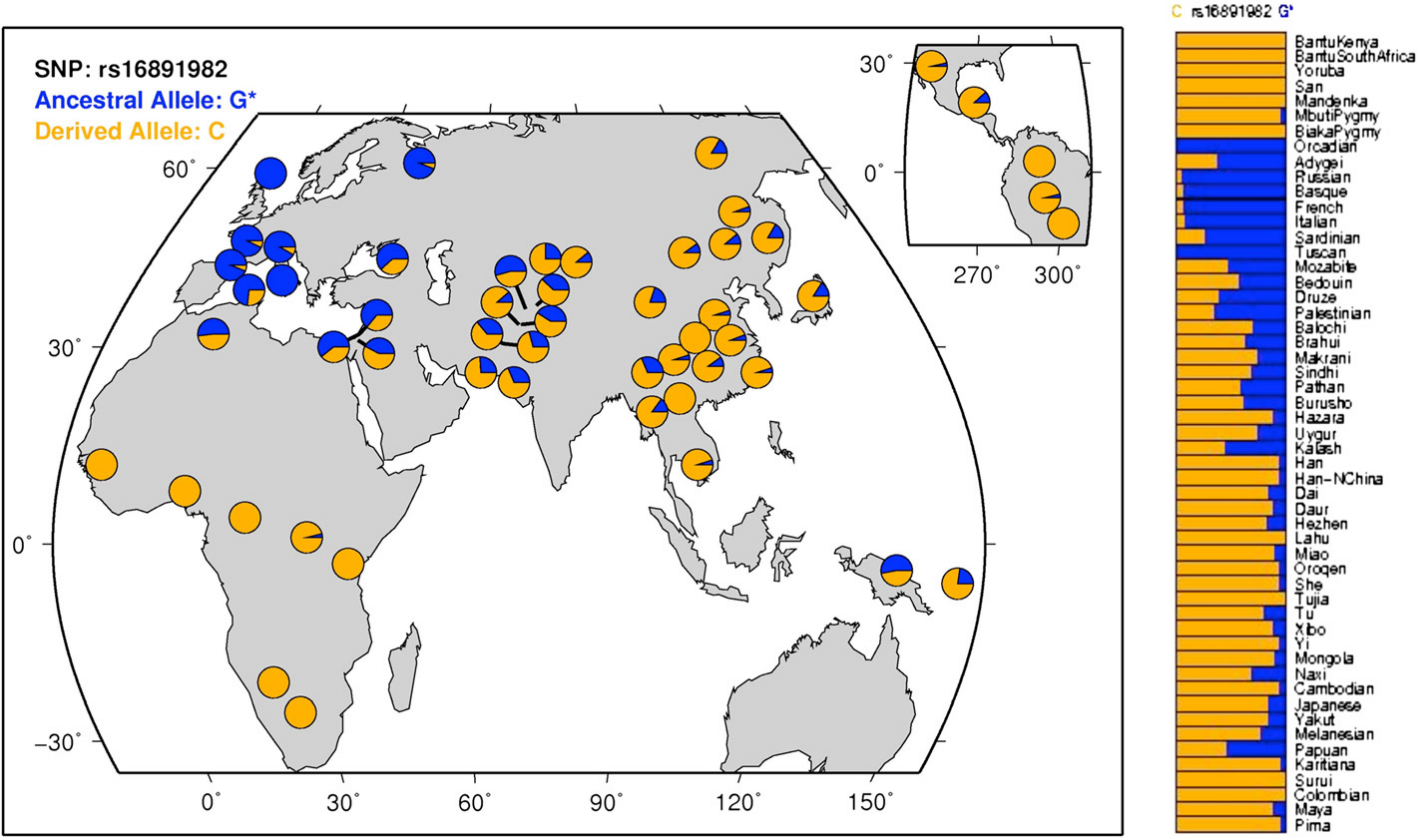
**HGDP population ancestral allele frequency distributions for the rs16891982 SNP **[16]**.** The distribution trends across the populations are analogous to those of the continents in Figure 4.

Our results emphasize both the genetic and geographic significance of the rs16891982 SNP across the world. Each of the 193 countries was mapped to a HGDP population and its corresponding continent. We plotted the distribution of the ancestral allele frequency for each HGDP continent by aggregating the allele frequencies of all the countries within a particular continent (Figure 4). The distribution magnitudes and trends were consistent with the allele frequencies of the SNP throughout the world when plotted by individual populations (Figure 5) [15]. For example, Figure 4 shows that the ancestral allele frequencies were the lowest for countries in Africa, but were highest across Europe. This indicates that the proportion of the risk allele is significantly higher in the European continent, since the ancestral allele matches the risk allele for the rs16891982 SNP. We would thus expect European populations to be more susceptible to melanoma, as compared to populations in continents with lower ancestral allele distributions. These results are supported by the incidence and mortality rates for melanoma published by the Center for Disease Control and Prevention (CDC) and other sources [18-20].

To an extent, worldwide variations in ancestral allele frequency distributions for the rs16891982 SNP may be explained by the disparity in UV radiation levels across different countries, as previously illustrated in Figure 2. Geographic regions with higher UV exposure, such as Africa and South America, have countries with a lower proportion of the ancestral allele. The derived allele *C* is more predominant in these regions. Evolutionary adaptations to UV radiation may account for the lower AAFs in these parts of the world, and thus lead to a lower susceptibility to cancers such as melanoma. The European populations, on the other hand, receive significantly lesser radiation and have higher AAFs. The negative correlation between AAF and UV radiation for this SNP was shown earlier in Figure 3.

In addition to the rs16891982 SNP, we also incorporated the complete pool of radiation SNPs in our analysis for selection. We developed an algorithm to identify the HGDP populations under positive selection for these SNPs. The algorithm utilizes systematic random sampling to calculate empirical p-values for each population, providing a measure for the significance of selection. The results of our simulation are shown in Table 2, in which we only focus on populations with an empirical p-value lower than 0.05. Eight distinct populations in Northern and Southern Europe, Russia, Israel, and Northern Africa were found to be under strong positive selection for large numbers of radiation SNPs. Taken collectively, the significant positive selection in these eight populations is analogous to the higher ancestral allele frequency distributions of the rs16891982 SNP in the corresponding geographic areas.

**Table 2:** Native populations under strong positive selection for radiation SNPs

**HGDP population**	**Area**	**SNP Count**	**Average iHS score**	**Empirical p-value**
Russian	Russia	413	0.99	2.1 × 10^-6^
French	Southern Europe	411	0.97	1.5 × 10^-6^
Basque	Southern Europe	375	0.96	1.4 × 10^-5^
Sardinian	Southern Europe	385	0.90	3.7 × 10^-4^
Orcadian	Northern Europe	397	0.89	6.5 × 10^-4^
Tuscan	Southern Europe	360	0.85	1.9 × 10^-2^
Druze	Israel	326	0.86	3.9 × 10^-2^
Mozabite	Northern Africa	304	0.85	4.6 × 10^-2^

### Gene ontology terms of radiation SNPs

To gain further insight into the relationship of UV radiation SNPs with human genetic functions, we have associated the 2,857 radiation SNPs with a derived gene function database from the Gene Ontology Consortium. We identified the genes in which the radiation SNPs were significantly enriched, by joining the gene list described in the methodology section with a table containing the ontology terms for these genes.

After a Bonferroni correction was carried out, the radiation SNPs were found to be significantly enriched in two gene ontology categories, with a 2-fold enrichment in genes for calcium ion binding (Pearson p = 2.3 × 10^-5^, corrected p = 0.04) and a 13.6-fold enrichment in genes for glutamate receptor activity (Pearson p = 1.73 × 10^-5^, Corrected p = 0.03), as shown in Table 3. The fold values were calculated using a Hypergeometric function.

**Table 3:** Ontology of genes in which the radiation SNPs were significantly enriched

**Gene ontology term**	**Gene count**	**Fold**	**Pearson p-value**	**Corrected p-value**
Glutamate receptor activity	5	13.6	1.7 × 10^-5^	0.031
Calcium ion binding	40	2.0	2.3 × 10^-5^	0.041

After performing a Bonferroni correction, genes with a p-value under 0.05 were further studied for relation to UV radiation.

Overall, the radiation SNPs were robustly enriched in calcium ion binding genes throughout all analyses using Pearson’s rho cutoffs ranging from 0.70 to 0.85. This is consistent with published studies indicating that UV irradiation of lymphocytes induced calcium flux and tyrosine phosphorylation [21]. It was previously shown that rapid calcium responses were directly induced in both dose- and wavelength-dependent manners. Genes involved in the activity of glutamate receptors were only enriched when a Pearson’s rho cutoff of 0.8 was used.

## Discussion and conclusion

In this paper, we presented a systematic approach for both identifying DNA variants associated with UV radiation and for studying their connections to diseases such as skin cancers, via a novel geographic-wide association study. Our analyses have led to a better understating of why certain diseases are more predominant in particular geographical locations, due to the interactions between environmental variables such as UV radiation and the population genetics in those regions.

Taken collectively, our results have both biomedical and methodological significance. From a biomedical context, we were able to identify SNPs that had AAFs strongly correlated with UV exposure levels across the world. From the pool of radiation SNPs, we identified candidate SNPs that were enriched for association with different diseases and phenotypes, most of which had a direct link with UV radiation. We found an enrichment in genes associated with calcium ion binding and glutamate receptor activities. One potential explanation for these findings is that UV radiation causes an excess of glutamates to accumulate in the extracellular space, resulting in an influx of calcium ions into the cytosol and thus increasing the risk of brain disorders. The process in which high levels of calcium ions enter cells through glutamate receptors, ultimately causing apoptosis, is called excitotoxicity [22]. Excitotoxicity is known to be associated with neurodegenerative diseases such as multiple sclerosis, Alzheimer’s disease, and Parkinson’s disease.

As a case study, we chose the rs16891982 SNP to analyze its association with melanoma, using its allele frequency distributions in the HGDP populations to explain why certain groups of people in the world are more susceptible to melanoma, and how natural selective pressures from environmental factors such as UV radiation may have played an important role. The findings were consistent with published data for the worldwide incidence and death rates for melanoma. We also devised an algorithm to calculate the degree of positive selection for radiation SNPs in native populations worldwide. From the same group of SNPs, we determined the ontology of genes in which the SNPs were significantly enriched, surveyed known relationships in literature, and can propose hypotheses to explain our observed findings at the genetic level.

From a methodological standpoint, we have developed a powerful approach that extends the current GWAS model (Genome-Wide Association Studies) to incorporate countries into the analysis. The algorithm designed in this study integrates worldwide SNP and UV data to simultaneously compare the risks of different diseases across the world. Using again the case study of the radiation SNP rs16891982 as an example, we were able to expand earlier approaches that only accounted for single countries [5,6] by systematically identifying SNP-disease associations for different HGDP populations on a global scale. Unlike former studies that used pre-selected SNPs, the GeoWAS algorithm identifies all possible SNPs in a more efficient and objective manner.

The GeoWAS methodology presented in this paper can also be applied to other long-term environmental variables such as temperature, precipitation, and humidity. The algorithm can be similarly used to carry out analyses of the genomes of other species. Furthermore, the UV radiation SNPs identified in this work may potentially be used in aiding skin cancer diagnostics, through *in vitro* methods based on the expression of the *SLC45A2* gene [23].

With our developed methodology, we were able to investigate the effects and implications of UV radiation on human phenotypes and diseases at both the genomic and geographic levels. The significance of GeoWAS and its promises are discussed from both biomedical and methodological perspectives. Ultimately, the GeoWAS approach can benefit epidemiologists, and physicians, and policy-makers in regions of the world where conventional clinical studies are either costly or unavailable. Future work will involve refining the current approach by performing the country-population mappings based on updated HGDP coordinate systems.

## Competing interests

Atul Butte is a founder of Personalis, Inc., which has acquired a license to VARIMED from Stanford University. Rong Chen is now an employee of Personalis, Inc.

## Authors’ contributions

IH and RC contributed equally to this work and carried out the analysis. IH performed the research, designed the case study, and wrote the manuscript. RC first proposed the problem to be addressed. AJB and RC conceived the study and provided input on methodology throughout. AR and EC provided expertise in algorithm development for positive selection. PK contributed to analysis of SNPs in Vitamin D synthesis. DR supplied statistical expertise and input on research. All authors read and approved the final manuscript.

## Pre-publication history

The pre-publication history for this paper can be accessed here:

http://www.biomedcentral.com/1471-2350/14/62/prepub
